# Epidemiological, Clinical, and Laboratory Characteristics of Acute Disseminated Encephalomyelitis in Children: A Retrospective Study

**Published:** 2019

**Authors:** Mohammad Mahdi TAGHDIRI, Masoud Hassanvand AMOUZADEH, Shaghayegh Sadat ESMAIL NEJAD, Ezatollah ABASI, Abbas ALIPOUR, Mohsen AKHAVAN

**Affiliations:** 1 Pediatric Neurology Research Center, Research Institute for Children’s Health, Shahid Beheshti University of Medical Sciences, Tehran, Iran; 2 Pediatric Neurology Department, Mofid Children’s Hospital, Faculty of Medicine, Shahid Beheshti University of Medical Sciences,Tehran, Iran; 3 Neuroscience Research Center, Qom University of Medical Sciences, Qom, Iran; 4 Pediatric Department, Faculty of Medicine, Urmia University of Medical Sciences, Urmia, Iran; 5 Department of Epidemiology, Faculty of Health, Shahid Beheshti University of Medical Sciences, Tehran, Iran; 6 Department of Pediatric Nephrology, Faculty of Medicine, Qom University of Medical Sciences, Qom, Iran

**Keywords:** Acute disseminated encephalomyelitis, Epidemiology, Magnetic resonance imaging, Children

## Abstract

**Objectives:**

We aimed to study the precipitating factors, demographic data, clinical and radiological manifestations, electroencephalography and laboratory findings, as well as association with infections, immunization and incidence of relapse of acute disseminated encephalomyelitis (ADEM) in children admitted to Mofid Children Hospital, Tehran, Iran from Mar 2013 to Mar 2016.

**Materials & Methods:**

A 3-yr retrospective review of 29 children with definite final diagnosis of ADEM in Mofid Hospital in Tehran, Iran was performed. The diagnosis was based on specified criteria, including a presumed acute demyelinating process with no history of unexplained neurological symptoms and at least one demyelinating lesion shown on magnetic resonance imaging without evidence of previous destructive white matter lesions.

**Results:**

Overall, 29 children diagnosed as ADEM were studied in terms of demographic characteristics, clinical manifestations and laboratory findings in two groups according to their recurrence. The mean age of the patients with recurrence was less than those without it were. It was more common in females but the difference was not statistically meaningful. There was no relationship between the season of the first episode of the disease and the recurrence incidence. Moreover, the relationship between viral infections and recurrence was statistically non-meaningful. No relationship between the recurrence of ADEM and clinical manifestations, radiological and laboratory findings was found.

**Conclusion:**

The reason for high rate of recurrence in our patients may be related to the younger age of children in our study.

## Introduction

Acute disseminated encephalomyelitis (ADEM) is a kind of inflammatory demyelinating disease of the CNS which usually occurs secondary to an antecedent infection or immunization. Although the pathophysiological mechanism of ADEM is unknown, it is thought to be a T-cell immune-mediated response to myelin basic protein triggered by infections or vaccination. The clinical manifestations are different in each patient characterized by an acute or subacute onset of multifocal neurologic disturbances that typically follow a monophasic course in which a wide range of neurological and non-neurological signs and symptoms has been reported such as fever, malaise, myalgia, headache, nausea and vomiting, meningismus, convulsions, cranial nerve palsies, ataxia and psychosis (1-9). 

ADEM is a kind of neurological disease, which predominantly affects children and young adults. The diagnosis may become difficult because in 30% of cases, the first episode of ADEM subsequently evolves typical multiple sclerosis (MS) (8). The occurrence of recurrent periods and relapses in some of the patients make it difficult to distinguish from MS (2,9). Although recurrence may occur, the prognosis is usually good and most of the children make a full recovery (6).

ADEM may occur in any age but it is often seen in average age of 5 to 8 yr and is mostly in male (15). 

The definite diagnosis is not available by a kind of pathognomonic clinical or laboratory findings and patients usually has a normal brain computed tomography (CT) features. Magnetic resonance imaging (MRI) by showing high signal lesions of the same age on T2-weighted images will help us to make the diagnosis of ADEM. High signal lesions are usually seen in the subcortical white matter but there may be some lesions in the cerebellum, grey matter, basal ganglia, periventricular regions, brain stem, thalamus, midbrain or spinal cord (6,8,16,17).

Prescribing high dose methylprednisolone, dexamethasone, intravenous immunoglobulin (IVIG) and plasmapheresis are used to manage the patients diagnosed as ADEM (6,18-20). The increasing rate of using MRI as a brain imaging in recent years has led to more correct diagnosis of ADEM in children which results in more effective treatments (6,21).

“The exact incidence of ADEM is not known but it has been reported approximately 0.4/10 ^5^/year among people less than twenty years old in San Diego County” (22). ADEM was more common in children population because of higher exposure to antigens and higher frequency of immunization and viral infections in children (9). Most of the studies about ADEM patients have been done on pediatric populations reported mostly in Caucasians (22).

Few large series of ADEM have been published in Asian populations (22-24), and similar studies are scarce in Iran. Thus we tried to study the precipitating factors, demographic data, clinical and radiological manifestations, electroencephalography (EEG) and laboratory findings, association with infections and immunization and incidence of recurrence in the children diagnosed as ADEM admitted to Mofid Children Hospital, Tehran, Iran.

## Materials & Methods

From Mar 2013 to Mar 2016, 36 patients with initially diagnosed as ADEM were admitted to Mofid children Hospital, Tehran, Iran. Their medical records were reviewed retrospectively and 7 cases were excluded with different diagnoses such as MS, CNS infection, isolated transverse myelitis, optic neuritis, etc.

Overall, 29 cases were entered our case-series study with the definite diagnosis of ADEM. Moreover, telephone interviewing was done carefully for each case in order to complete the data which was all classified in a chart questionnaire. 

The data was recorded confidentially and informed consent was obtained from parents. Each person was characterized by a numerical code.

ADEM diagnosis was made by the criteria, including a presumed acute demyelinating process with no history of unexplained neurological symptoms and at least one demyelinating lesion shown on MRI without evidence of previous destructive white matter lesions (25).

The demographic, clinical, laboratory and radiological data of each case were reviewed in detail, including sex, age, season of admission and its duration, any history of antecedent infectious disease specially viral infections, history of vaccination during last month, clinical manifestations, EEG findings, exact region of CNS lesion shown by MRI, CSF analysis, CBC (complete blood count), ESR, qualitative CRP. Moreover, recurrence and the time between first onset and first recurrence was asked.

CSF analysis was performed within first 2 days of admission in the majority of patients included WBC count, cytology, glucose and protein concentration, CSF culture and microbiological investigations.

Almost all of the cases underwent brain and spinal MRI study in the first days of admission except for those who had loss of consciousness and ICU admitted. MRI and EEG studying were performed after clinical stabilization and were reported by an expert neuroradiologist and pediatric neurologist, respectively. 

The clinical manifestations including the early onset presentations were recorded, in addition to the signs and symptoms presented during the time of admission.

The laboratory studies were all done in the same laboratory and abnormal findings were all rechecked.

The data were analyzed by SPSS (ver. 22.0, Chicago, IL, USA) Statistical software.

## Results

Twenty-nine children out of 36 cases diagnosed as ADEM were enrolled and were studied in terms of demographic characteristics, clinical manifestations and laboratory findings classified according to the recurrence (Table 1). Table 2 shows clinical manifestations, EEG and MRI findings and Table 3 shows the laboratory findings.

In 9 patients (31%) recurrence occurred in which the mean period of time between the recovery after the first onset of the disease and its recurrence was 5.2 months. ADEM recurrence incidence rates per 1000 person-months were 17.41 (95% CI: 9.6-33.46). None of the patients have family history of ADEM. 

The mean age of the patients with recurrence was less than those without it were. It was more common in female population than male but the difference was not meaningful in terms of statistical studies.

There was no relationship between the season of the first episode of the disease and the recurrence incidence (*P*=0.47). None of the children who showed recurrent episodes had history of vaccination but 4 cases of the group without recurrence had done immunization in the last month before the first onset of disease. Of course, the difference was not statistically meaningful (*P*=0.28). The viral infection was reported in 4 children with recurrence and 9 without. The statistical studies also showed no meaningful difference (*P*=0.99).

No relationship between the recurrence of ADEM and clinical manifestations, radiological and laboratory findings was found.

**Table 1:** The epidemiological characteristics of the patients according to the recurrence

Variables		Recurrence		Total (N=29) n (%)	*P*-value
		No (N =20) n(%)	Yes(N =9) n (%)		
Age (month) Mean(standard deviation)		57.3(47.02)	53.33(31.65)	56.07(42.3)	0.87
Sex (female/male)		7/13	6/3	13/16	0.23
Season	Spring	6(30)	1(11.1)	7(24.1)	0.47
	Summer	5(25)	3(33.3)	8(27.6)	
	Autumn	5(25)	3(33.3)	8(27.6)	
	Winter	4(20)	2(22.2)	6(20.7)	
History of viral infection		9(45)	4(44.4)	13(44.8)	0.99
History of vaccination		4(20)	0(0)	4(13.8)	0.28

**Table 2:** Clinical manifestations and the findings of EEG and MRI studies

Variables		Recurrence		Total(N=29) n(%)	*P*-value
		No(N =20) n(%)	Yes(N =9) n(%)		
Clinical manifestations	Motor deficit	8(40)	5(55.6)	13(44.8)	0.69
	Consciousness alteration	8(40)	3(33.3)	11(39.7)	0.99
	Headache	2(10)	0(0)	2(6.9)	0.99
	Seizure	9(45)	4(44.4)	13(44.8)	0.99
	Ataxia	5(25)	3(33.3)	8(27.6)	0.68
Lesions shown in MRI	Periventricular	3(15)	0(0)	3(10.3)	0.53
	Brain stem	3(15)	1(11.1)	4(13.8)	0.99
	Cerebellum	1(5)	2(22.2)	3(10.3)	0.22
	Basal ganglia	1(5)	1(11.1)	2(6.9)	0.53
	Cortex	4(20)	1(11.1)	5(17.2)	0.99
	Subcortical white matter	12(60)	3(33.3)	15(51.7)	0.25
	Thalamus	2(10)	0(0)	2(6.9)	0.99
	Spinal cord	2(10)	2(22.2)	4(13.8)	0.57
	Midbrain	0(0)	1(11.1)	1(3.4)	0.31
Abnormal EEG		9(45)	6(66.7)	15(51.7)	0.43

**Table 3:** Laboratory findings

Variables	Recurrence		Total (N=29) Mean(standard deviation)	*P*-value (Mann Withney U test)
	No (N =20) Mean(standard deviation)	Yes (N =9) Mean(standard deviation)		
CSF leukocyte count	3.25(9.96)	0(0)	2.48(8.74)	0.45
CSF protein	19.25(11.07)	21.8(9.04)	19.86(10.46)	0.40
CSF glucose	68.5(25.55)	61.6(11.28)	66.86(22.9)	0.72
ESR	17.4(20.31)	30.25(20.37)	20.11(20.47)	0.19
Blood WBC count	7900(3984.53)	8455.56(2123.15)	8085.2(3440.8)	0.60
CRP n(%)	7(50)	2(28.6)	9(42.9)	0.40

## Discussion

Our study is one of the few types of research, which have reviewed epidemiological, clinical and laboratory data of children with ADEM in two categories: with or without recurrence. The mean age of the patients in our study was 56.07 months which was less than other studies (over 5 yr old) (26-30). Male/ female ratio in our study was 1.23 which was consistent with previous studies (28,29,31).

In our study, the seasonal distribution of ADEM was equal, while in previous studies the most incidence of ADEM was in winter (10,32,33). Seasonal priority in those studies may be justified by more incidence of febrile viral infections in winter incriminated for ADEM.

44.8% of the patients in our study had a history of a febrile infection last month before ADEM presentations but this amount was different in other studies (more than 70%) (4,5,8,10,27,29,32,34,35). The current result of our study is consistent with the last study in Iran (46.4%) (9). History of febrile disease (mostly viral infections) has a small causal role in the incidence of ADEM in our country. Only 13.8% of the patients in our study had a history of vaccination in the last month before presentation of the disease which was the same as other studies (9, 29,35). 

The most common region of the brain lesions was subcortical This matter according to our study (51.7%) This result was similar to previous studies; Though, the rate of sub cortical white matter involvement in the preview studies (more than 80%) (9,10,29,32,34).

Motor deficit and seizure were the most common signs in our patients with the incidence rate of 44.8% separately, and consciousness alterations, ataxia, and headache were the next common signs, respectively. In previous studies motor deficit was also the most common sign; Ataxia and consciousness alterations were reported as most common signs after motor deficit (4,6,9,23,26,34). These results showed a more incidence of seizure in our study.

In our study, almost half of the patients had abnormal EEG findings (51.7%) which was less than other studies (more than 70%) (4,5,29,32). The less amount of EEG abnormalities in our patients despite the more incidence of seizure shows that the most common cause of seizures was acute cerebral injury which does not necessarily need long time medical therapy.

In our research lumbar puncture was performed in 22 cases. The mean leukocyte count was 2.48 cell/µl, CSF protein was 19.86 mg/dl and CSF glucose was 66.86 mg/dl, all showed normal values. CSF protein was in normal range in all cases, while CSF glucose was less than normal range in only one patient and the CSF leukocyte counts were abnormal in two of them. These findings were quite in contrast to other studies reported CSF abnormalities (leukocyte count, protein or glucose concentrations) in more than two-thirds of the patients (4,9,10,22,24,29,31,32). This result may be attributed to the possible role of viral infections in ADEM.

In our study, 19 patients had ESR results in their laboratory data in which mean amount of ESR was 20.11 mm/hour and was consistent with previous studies (23, 32).

Laboratory data of 27 cases included CBC with average count of 8085 cells/µl and only two of them had leukocytosis. This was in contrast with other studies reported leukocytosis in one third to two-thirds of the patients (6, 23, 29, 32). The leukocytosis in these studies may be justifiable with the possible role of viral infections in ADEM.

Laboratory data of 21 cases included CRP which was positive in 42.9% of them. In previous studies, CRP was reported normal in approximately all of the patients (5, 23, 29). Positive CRP reported in our study may be due to the fact that CRP is an acute phase reactant accompanies inflammation and tissue injury.

In our study after a mean follow-up period of 23.1 months (range: 6-37 months), 9 cases (31%) showed recurrence and no death was reported. In previous studies, the recurrence rates were reported less than ours (10%-15%) (5,6,32,34). 

Although in our study the epidemiological, clinical and laboratory data of the patients were reported separately in two groups of patients divided in with or without recurrence, no meaningful statistical difference was found between two groups. 

**In conclusion,** compared with previous studies, the reason for high rate of recurrence in our patients may be related to the younger age of patients in our study. Finally, we suggest more comprehensive studies in order to find the underlying causes of high rate of recurrence of ADEM in Iranian children.
